# Changes in opiate and stimulant use through 10 years: The role of contextual factors, mental health disorders and psychosocial factors in a prospective SUD treatment cohort study

**DOI:** 10.1371/journal.pone.0190381

**Published:** 2018-01-25

**Authors:** Grethe Lauritzen, Trond Nordfjærn

**Affiliations:** 1 Department of Drug Policy, Norwegian Institute of Public Health, Oslo, Norway; 2 Department of Psychology, Norwegian University of Science and Technology, Trondheim, Norway; 3 Department of Research and Development, Clinic of Substance Use and Addiction Medicine, St. Olavs University Hospital, Trondheim, Norway; Monash University, AUSTRALIA

## Abstract

**Aim:**

To examine temporal changes in opiate and stimulant use among patients in substance abuse treatment over a ten-year observation period and to explore the role of contextual factors, mental health disorders and psychosocial factors on these changes.

**Methods:**

A cohort of 481 patients was prospectively interviewed at admission to treatment and after 1, 2, 7 and 10 years. The sample was recruited from 20 facilities in the Greater Oslo region, Norway.

**Results:**

The majority of patients were poly-drug users and 80% had used both opiates and stimulants the last 30 days prior to treatment admission. Last-month use of heroin, other opiates, cocaine and amphetamines declined from 80% to 34% at the end of the observation period. The most substantial reduction was observed between baseline and one-year follow-up. Use of heroin decreased the most from 62% to 16% after 10 years (a reduction of 74%), and the reduction continued from one-year follow-up throughout the observation period. The most important multivariate risk factors for sustained use of these drugs were male gender, having one or both biological parents with severe alcohol or drug problems, having an antisocial personality disorder, and living together with a person who abuses alcohol or drugs. Employment was associated with reduced risk of drug use at 7-year follow-up.

**Conclusions:**

There was a substantial reduction in opiate and stimulant use from baseline to all follow-up assessments, most greatly for heroin. Findings regarding sustained use could suggest familial transmission and the challenges of preventive strategies and treatment efforts in an intergenerational context. Co-occurrence between drug abuse and mental health problems highlights the need of highly specialized competence in SUD treatment.

## Introduction

A strong focus is warranted on outcomes among patients in substance use disorder (SUD) treatment. SUD creates substantial suffering both for the individual and significant others, premature death and huge societal costs. Clinical observations and treatment studies have shown that SUD tends to be a persistent condition, often with need for long trajectories of treatment efforts to obtain drug-free and/or good quality lives [1], [2]. Long-term observations with an overriding aim of exploring the fluctuations and life cycles of drug use along with treatment episodes are scarce. The current prospective study was conducted to follow opiate and stimulant drug use courses during 10 years in a cohort of illicit drug users recruited from Norwegian treatment facilities. The focus was to specifically explore changes in the use of these two categories of drugs due to their high-risk potential of dependence, severe physical and mental harm and deadly overdose potentials. The study also set out to identify predictors of change in use of these drugs throughout the observation period. The current research extends a previous study applying the same data material to examine use of drugs in relation to mental distress [3].

Several prospective cohort studies have been carried out among patients with SUD. The largest cohort studies were initiated in the United States. The Drug Abuse Reporting Program (DARP) [4] followed more than 4000 patients through three years. The Treatment Outcome Study (TOPS) [5] interviewed three cohorts recruited from treatment, whereof one was followed up after 1 and 2 years, the second cohort after 3 and 12 months and the last cohort from 3 to 5 years. The Drug Abuse Treatment Outcome Study (DATOS) [6], [7] followed patients during treatment and after 1 and 5 years. These studies found consistent decrease in drug use 1-year post-treatment as well as within a 5-year period. The studies focused on changes in use of single drugs, mainly heroin, and pointed at the completion and the temporal length of treatment as predictors for reduced drug use [8], [9].

The National Treatment Outcome Research Study (NTORS) [10], [11], [12] was a prospective study conducted in the United Kingdom among 418 patients interviewed at intake to treatment, and after 1, 2 and 4–5 years. The results were consistent with findings reported in the United States and showed that the greatest decline occurred within the first year, but with levels of use tending to stabilise or declining at 4–5 years. The Australian Treatment Outcome Study (ATOS) [13], [14], [15], [16], [17] included a cohort of 615 heroin users interviewed at 3 months and followed up after 1, 2, 3 and 11 years. A substantial reduction in heroin use was observed from baseline to the two-year assessment (99% vs 35% use last 30 days) and a stable rate was found in the post-treatment assessments. Reductions in drug use were also here associated with length of time in residential treatment and to fewer additional treatment episodes. Factors associated with sustained heroin use in previous work were generally shorter retention times [13], [18], [6], [2], a history of previous treatment [19] and more treatment episodes over the follow-up period [18], [13].

All of the studies cited above draw attention to the amount of drug reduction during the first year, which may be attributed to baseline treatment factors and could reflect a dose-response relation with treatment. The relatively short follow-up periods and few assessment waves limit conclusions regarding long-term drug-use trajectories in previous research. These studies also tended to investigate changes and outcomes in single-drug use. There seems to be a gap in the literature regarding longitudinal patterns of poly-drug use, even if this appears to be a highly common use pattern among patients with SUD [20], [21]. The current study, with its long observation period and focus on poly-drug use; i.e. heroin, other opiates, cocaine and amphetamines, is an effort to address this limitation in the literature. The primary research questions of the study were: How did this combination of drugs change during 10 years and what predictors could be identified for temporal changes in use of this poly-drug combination?

Previous work in this vein tended to mainly focus on factors concerning additional treatment and client-centred factors such as socio-demographic characteristics, childhood trauma, and personality disorders/psychological problems. In addition to these variables we also focus on substance abuse among the respondents’ biological parents. This approach was based on the theoretical and empirical intergenerational perspectives on drug use, explained mostly as a complex interplay between genetic and environmental factors [22], [23], [24]. To our knowledge such factors have not been focused in previous longitudinal studies. Further, it has been argued in theoretical frameworks, such as the behavioural choice theory [25] that contextual and psychosocial factors are critical in relation to drug use outcomes among patients with SUD. Decades ago, Robins et al. also underlined the role of these variables [26], but surprisingly few studies have actually focused on these beyond the mere adjustment for them as confounds in statistical analyses.

In the current study, we grouped the predictors of temporal changes in opiate and stimulant use into contextual factors, mental health disorders and psychosocial factors. Contextual factors were operationally defined as work income, housing, living with a person who has substance abuse problems and additional treatment measured at each assessment wave. Other contextual factors were gender, age and level of education. Mental health disorders included the occurrence of anxiety, depression and personality disorders, whereas psychosocial factors covered childhood trauma and severe substance abuse and mental health problems among biological parents. Some of these factors have been described as potential causes and moderators of drug abuse and SUD in the international literature [15], [6]. In particular, the mental health disorders were chosen in line with previous studies. Examining contextual variables, mental health disorders and psychosocial variables in tandem allows us to investigate their relative roles for changes in opiate and stimulant use. To our knowledge, no studies have investigated multivariate prospective associations between these three categories of factors with changes in use of opiates and stimulants over a lengthy temporal period using multiple measurement waves.

The aims of this 10-year prospective study are to investigate temporal changes in opiate and stimulant use and to examine the role of contextual factors, mental health disorders and psychosocial factors for these changes in a SUD treatment cohort.

## Material and methods

### Procedure

#### Study design

The study was conducted with a naturalistic prospective cohort design and the observation period was 10 years. Twenty treatment facilities were purposively selected for patient recruitment. The facilities represented the most common treatment programmes in Norway; (residential treatment, specialised therapeutic outpatient teams and Opioid Maintenance Treatment (OMT)) (see also [27], [28], [29]). The chosen geographical area for recruitment of facilities was the capital of Norway and surroundings (i.e., the greater Oslo region), where these treatment modalities were available and the illicit drug problems are the most prevalent. Data were collected by face-to-face interviews and by self-report instruments (within the same encounter). The baseline interview was accomplished within the first two weeks of treatment, and the follow-ups were scheduled at 1, 2, 7 and 10 years later. The study protocol was approved according to existing standard procedures by the Norwegian Social Science Data Service (NSD) (97/3536) and the Norwegian Data Inspectorate (97/3536) before the study started. There were no relationships or conflicts of interest between the participants and the research team.

#### Sampling

A total of 481 patients were recruited on treatment entry in the period January 1998–August 2000 (baseline), and the inclusion criteria were: (a) enrolment in one of the 20 units and (b) needing treatment for *illicit* drug use. All patients who met these two criteria were asked to participate in the study. Written informed consent was obtained from all participating patients. Most of the treatment units were rather small and the patient turnover limited due to long-term treatment. Of the 481 patients who participated at baseline, 307 (64%) were recruited from residential units, 100 (21%) from therapeutic outpatient teams and 74 (15%) from OMT. The participation rate at baseline was 93% of the sampling frame in the residential units and 76% of the sampling frame in the OMT teams. It was difficult to calculate an exact response rate from the therapeutic outpatient teams because of a more flexible intake procedure and an initial patient drop-out from the teams [29]. There were no substantial differences in gender, age and drugs used at baseline between participants and non-participants [29].

Of the 481 respondents at baseline, 428 were interviewed at the 1-year follow-up, 410 after 2 years, 348 after 7 years, and 296 after 10 years. Cumulatively, there were 11 (2.40%), 19 (4.20%), 59 (13.10%) and 72 (16%) deceased at the four follow-up interviews. Some differences between the deceased and non-deceased patients were found [28]. The patients who died during the follow-up years had a slightly higher number of nonfatal overdoses prior to index treatment; they had more years with alcohol abuse and spent longer time in prison than the non-deceased. Males had 2.80 times higher probability than females of dying in the course of the observation period. The retention rates for the follow-up waves were 91%, 89%, 85% and 77% when the deceased were included. Fifty-four percent (n = 260) participated in all four follow-up interviews. The 260 patients who participated across all measurement waves did not differ significantly from the group with 1–4 follow-ups on baseline demographic variables, mental health measures or drug use except for a slight difference in use of methadone (p < .05), showing somewhat fewer of the patients attending all follow-ups using methadone at time of inclusion.

### Measures

#### Opiate and stimulant use

Drug use was measured with the European adaptation of the Addiction Severity Index (EuropASI) [30], [31] and we utilized a Norwegian version of the EuropASI, translated/back-translated and approved by the Kokkevi/Hargers group [30]. Questions about heroin, other opiates, cocaine and other amphetamines were worded: “How many days during the last 30 days have you used …”. The variables were dichotomized to a no use/use response category at each assessment wave.

#### Contextual factors

Information about gender, age, education, work income and living together with a person abusing alcohol or drugs was taken from the EuropASI. Completed high school education was used as a cut-off for “high education”. In addition to these contextual factors, we also included the three ASI categories of treatment: Inpatient, OMT, and outpatient-therapeutic teams. A >0 response to the question: “how many sequences of treatment have you had since the last interview in the observation period?” was used for assessing additional treatment between the interview waves.

#### Mental health disorders

We assessed psychiatric symptoms (Axis I) and personality traits (Axis II) with the self-report Millon Clinical Multiaxial Inventory II (MCMI II) [32]. The instrument consists of 175 statements on which the respondents answer “right” or “wrong”. The scores on MCMI are reported as base-rate scores (BR), transformed raw-scores and adjusted for gender differences. A score of 84 and above is considered clinically significant. We investigated the clinical symptom scales of anxiety and dysthymia. Dysthymia was chosen to avoid Type II error inflation in analyses due to a very low prevalence of major depression (3%, n = 15).

#### Psychosocial factors

The Childhood Trauma Questionnaire (CTQ) [33], [34], [35] was used at baseline for assessing childhood maltreatment and traumas. The CTQ is a 28-item self-report inventory, which enquires about five types of maltreatment: emotional, physical and sexual abuse, and emotional and physical neglect. The current study used the three abuse scales to capture the most severe maltreatment and in order to avoid multicollinearity in analyses. The individuals responded to statements about childhood circumstances with the following response options: “never true”, “rarely true”, “sometimes true”, “often true” and “very often true”. Thresholds or cut-off scores have been set for each type of trauma at four levels: None (or Minimal), Low (to Moderate), Moderate (to Severe) and Severe (to Extreme) [34]). We dichotomized the four levels into Low (None and Low) and High (Moderate and Severe) on the three scales of emotional, physical and sexual abuse. This was done to capture cases of moderate, severe and extreme abuse in one category of predictors against cases with no or low severe exposure of abuse. Furthermore, there were relatively few patients in the moderate and high response categories and merging these two reduced the likelihood of Type II error. In addition to the CTQ, information about learning and behavioural problems in primary school was also recorded.

Separate EuropASI questions for having had a mother or father who abused alcohol or drugs were collapsed to “one or both parents abused alcohol or drugs”. A similar method was used to group individuals who reported to have had a mother or father with considerable mental health problems. Both of these questions were worded: Have your mother/father had a substantial alcohol/drug problem which led to or should have led to treatment? Have your mother/father had a mental problem which led to or should have led to treatment?

#### Statistical analysis

IBM^®^ SPSS^®^ Statistics 22.0 was used to reveal characteristics of the sample, proportions of individuals within the contextual factors, mental health disorders and psychosocial factors as well as drug use during the previous month. Changes in drug use are described both by numerical differences and the corresponding percentages of change that these differences represent. McNemar’s tests were used to investigate whether changes in drug use were significantly different across the five measurement waves. Šidák corrections were applied to control for Type I error inflation. Before multivariate analysis was conducted the correlation matrix was inspected for potential multicollinearity between the predictor variables. To investigate potential multicollinearity in further detail the variance inflation factor (VIF) was inspected for each predictor. A cut-off value of 5.00 has been suggested to indicate multicollinearity [36]. We used STATA 13.1 to carry out logistic multilevel modelling and the models were tested with an unstructured covariance matrix, with random intercept and slopes (see also [37] for details). Multilevel modelling was chosen because more traditional logistic regression approaches tend to treat the respondents as independent cases. The current study consisted of a multilevel data structure with the same patients nested over multiple observation data points in time. Multilevel modelling is also more adequate when there are more than two follow-up waves and allows the inclusion of cases in analysis with missing data on one or more waves. This analysis was conducted by a two-step procedure; (1) we tested a model that only included temporal change in opiate and stimulant use over the five measurement waves, and (2) another model was tested where the contextual factors, mental health disorders and psychosocial factors were included as predictors of opiate and stimulant use. All predictors were entered as fixed covariates with the exception of work income, living with a person who abuses drugs, and enrolment into OMT or inpatient treatment during the study period. These factors were entered as interaction terms with time (i.e., time varying covariates). It was of interest to investigate whether changes in these variables were related to changes in drug use during the study period.

## Results

### Sample characteristics

At baseline the sample consisted of 68% males and the average age was 30.70 years (SD = 8.04) (Table 1). A total of 80% reported use of one or more of the categories of drugs; heroin, other opiates, cocaine and amphetamines during the last 30 days prior to index treatment. Heroin was the most frequently used drug, while only 5% reported use of cocaine (Table 1). More than 80% had injected drugs and approximately 60% reported one or several life-threatening overdoses during their lifetime. The sample was relatively poorly educated with less than one third having an educational attainment of high school level or above. Approximately one out of ten had a work-related income in the 30 days prior to inclusion and one fourth resided with a person who abused drugs. A total of 50% had previously been to inpatient treatment and one half had received treatment in specialised therapeutic out-patient teams. OMT was introduced in Norway around the same time as the study was initiated.

**Table 1:** Sample characteristics and univariate changes in drug use over time.

Indicator	Year 0	Year 1	Year 2	Year 7	Year 10
	*n* = 481(100%)	*n* = 428(89%)	*n* = 410(85%)	*n* = 347(72%)	*n* = 296(61%)
	*n* (%) or *M* (SD)				
*Contextual factors*					
Baseline treatment (yes)OMTInpatientOutpatient	74 (15%)307 (64%)100 (21%)	--	--	--	--
Age at baseline	30.70 (8.04)	--	--	--	--
Gender (male)	326 (68%)	286 (67%)	280 (69%)	231 (67%)	189 (64%)
Education (high)	134 (28%)	--	--	--	--
Income from work (yes)	55 (11%)	65 (15%)	81 (20%)	90 (26%)	87 (29)
Living with person who has substance abuse problems (yes)	122 (25%)	69 (16%)	61 (15%)	54 (16%)	38 (13%)
OMT intermediate^1^ (yes)	64 (13%)	79 (19%)	93 (23%)	148 (43%)	151 (51%)
Inpatient intermediate (yes)	251 (52%)	266 (62%)	184 (45%)	54 (16%)	35 (12%)
Outpatient intermediate (yes)	247 (51%)	142 (33%)	144 (35%)	162 (47%)	95 (32%)
*Mental health disorders*					
Anxiety (MCMI <84)	161 (34%)	--	--	--	--
Dysthymia (MCMI <84)	168 (35%)	--	--	--	--
Borderline personality disorder (MCMI <84)	129 (27%)	--	--	--	--
Antisocial personality disorder (MCMI <84)	250 (52%)	--	--	--	--
*Psychosocial factors*					
Parent(s) with psychological problems (yes)	171 (36%)	--	--	--	--
Parent(s) with alcohol or drug problems (yes)	224 (47%)	--	--	--	--
Primary school problems (yes)	306 (64%)				
Emotional abuse (M/H)	111 (24%)	--	--	--	--
Physical abuse (M/H)	99 (21%)	--	--	--	--
Sexual abuse (M/H)	130 (27%)	--	--	--	--
*Drug use past month (yes)*					
Heroin	299 (62%)^y1, y10^	128 (30%)^y0^	116 (28%)^y7^	56 (16%)^y2^	47 (16%)^y0^
Other opiates	106 (22%)^y1, y10^	62 (15%)^y0^	46 (11%)^y7^	64 (18%)^y2, y10^	30 (10%)^y0, y7^
Amphetamines	164 (34%)^y1, y10^	75 (18%)^y0^	67 (16%)^y7^	73 (21%)^y2^	55 (19%)^y0^
Cocaine	26 (5%)	21 (5%)	20 (5%)	19 (6%)	14 (5%)
Opiate and stimulant use	384 (80%)^y1, y10^	167 (39%)^y0^	159 (39%)	138 (40%)	101 (34%)^y0^

^1^Treatment prior to index treatment at baseliney = year

McNemar comparisons between y0 vs. y1, y1 vs. y2, y2 vs. y7, y7 vs. y10, y0 vs. y10.

Values with different subscripts are statistically different at p < .05 or below (Šidák corrected for multiple comparisons)

The self-reported psychosocial problems were comprehensive. Nearly 50% of biological parents of the patients had been in treatment, or were considered by the patient to be in need of treatment for substance abuse. Likewise, more than one third had received treatment (or according to the respondents needed treatment) for mental problems. The most prevalent type of childhood abuse was sexual abuse (27%). More than 60% reported primary school problems. Regarding mental health disorders, approximately one-third reflected symptoms of anxiety and dysthymia, while only 3% (n = 15) reflected major depression. One or more personality disorders were identified among 75% of the patients (MCMI-II, BR-score >84). The share with an antisocial personality disorder (ASPD) was the greatest, identified among 52%, and borderline personality disorder (BPD), one of the most severe personality disorders among 27%. About 40% reported having had one or more suicide attempts [3].

### Descriptive changes in opiate and stimulant use at the four follow-up assessments

There was a substantial decrease in the number of persons who reported use of the studied drugs from baseline to the end of the observation period. As shown in Table 1, the decrease was measured from a total of 80% reporting these drugs at baseline to 34% at the 10-year follow-up (a reduction of 58%) (McNemar’s χ^2^ = 110.41, p < .001). The greatest reduction was found between admission to index treatment and the 1-year follow-up (51%) (McNemar’s χ^2^ = 132.84, p < .001). The proportion of users was approximately equal at 1, 2, 7 and 10 years assessment after baseline. Of the four drugs, heroin use decreased the most, from 62% of the patients using at baseline to 16% at the 10-year follow-up (a reduction of 74%) (McNemar’s χ^2^ = 111.53, p < .001). Heroin use also had the strongest reduction between baseline and 1-year follow-up (McNemar’s χ^2^ = 104.01, p < .001), but continued to decrease from this point on to the 10-year wave (from 30% to 16%) (see Table 1 for details). Users of other opiates and/or amphetamines dropped respectively by 54%/44%, whereas the share of cocaine users was stable at the 5% level.

### Bi-variate associations between the study variables

Bi-variate correlations between the study variables are displayed in Table 2. The baseline predictors with the strongest cross-sectional associations with baseline opiate and stimulant use were inpatient index treatment and intermediate treatment as well as high age. The vast majority of predictors were weakly to moderately correlated, suggesting that multicollinearity was not a substantial issue. Some exceptions were observed among a few of the mental health disorders, with a rather strong positive correlation between dysthymia and anxiety. This aligns with the substantial comorbidity between these two diagnoses. However, none of the predictors had VIF values above the 5.00 cut-off.

**Table 2:** Bi-variate correlations between the main variables in the study.

Indicator	1	2	3	4	5	6	7	8	9	10	11	12	13	14	15	16	17	18	19	20	21	22	23	24	25	26
1.Baseline inpatient treatment	-	**-.68**	**-.57**	**-.17**	-.02	**-.12**	**-.23**	0.01	**-.22**	.10	**.12**	-.10	.03	-.03	-.05	-.09	-.02	-.02	-.06	.04	.06	**.14**	.07	.11	.05	**.17**
2. Baseline outpatient treatment		-	**-.22**	**-.21**	-.03	**.16**	**.39**	-.05	**-.13**	**-.31**	-.09	.05	-.07	-.02	.06	.00	-.03	-.02	.11	-.03	-.11	**-.33**	.07	-.07	-.05	**-.28**
3. Baseline OMT			-	**.47**	.06	-.02	-.12	.05	**.43**	**.21**	-.07	.08	.04	.04	.00	.12	.06	-.03	-.04	-.02	.05	**.18**	**.12**	-.08	.00	.09
4. Age				-	**.19**	.05	**-.17**	-.02	**.36**	.04	**.32**	-.06	-.02	-.10	.00	.10	.02	**-.13**	**-.15**	**-.18**	-.09	**.31**	.10	**-.17**	.00	**.19**
5. Gender (male)					-	-.06	-.04	**-.16**	.02	-.06	-.08	**-.12**	**-.13**	.03	-.05	.03	-.05	.05	.09	-.06	.03	.07	.02	.05	-.03	.03
6. Education (high)						-	**.18**	.01	.00	.04	.10	-.05	**-.15**	**-.17**	.06	-.01	.07	.03	.06	-.02	-.11	-.06	-.01	-.01	.10	-.08
7. Income from work (yes)							-	-.12	**-.15**	**-.19**	-.09	.03	-.04	-.11	.03	.03	-.10	.02	.03	.01	-.11	**-.17**	**-.16**	.00	.03	-.10
8. Living with person who has substance abuse problems								-	.05	.04	.05	.02	.09	.05	.02	-.01	.03	.03	-.01	.06	.06	-.01	-.01	-.10	.01	-.01
9. OMT intermediate									-	**.28**	.06	.01	.02	.05	.02	.03	.09	-.02	-.07	-.06	.04	.07	.10	-.08	.02	-.03
10. Inpatient intermediate										-	**.18**	.01	.05	.04	.03	-.02	.00	-.03	-.07	-.03	.02	**.29**	.11	.01	-.03	**.19**
11. Outpatient intermediate											-	-.12	-.02	.08	-.03	-.07	.02	.11	.05	.07	.06	**.14**	.04	-.01	-.03	**.14**
12. Parent(s) with psychological problems (yes)												-	**.25**	**.17**	.08	.12	.05	**.13**	.11	**.14**	.08	-.04	-.02	-.01	-.04	-.06
13. Parents(s) with alcohol or drug problems (yes)													-	**.18**	-.01	.07	.03	.04	-.02	.12	.08	.01	.03	.04	.01	.08
14. Primary school problems (yes)														-	.02	-.02	.04	.08	.07	**.14**	**.23**	-.01	.00	-.05	-.03	-.06
15. Emotional abuse (M/H)															-	**.42**	**.48**	.04	.04	.08	.08	.05	-.02	.00	-.02	-.01
16. Physical abuse (M/H)																-	**.28**	.04	.04	**.12**	.08	.06	.02	-.05	.06	.00
17. Sexual abuse (M/H)																	-	.05	.08	.05	.01	.06	.02	-.05	.00	**-**.04
18. Anxiety (MCMI <84)																		-	**.76**	**.52**	.11	-.08	.06	**.12**	.01	-.01
19. Dysthymnia (MCMI <84)																			-	**.57**	.08	-.09	.02	.08	.00	-.02
20. Borderline personality disorder(MCMI <84)																				-	**.33**	-.05	.05	.08	.04	-.03
21. Antisocial personality disorder(MCMI <84)																					-	.08	.00	.10	.05	.06
22. Heroin use last month (yes)																						-	**.22**	-.07	.02	**.64**
23. Oher opiate use last month (yes)																							-	.06	.05	**.27**
24. Amphetamine use last month (yes)																								-	**.14**	**.36**
25. Cocaine use last month (yes)																									-	**.12**
26. Opiate and stimulant use last month (yes)																										-

Significant correlations (p < .01) in bold

All indicators measured at baseline

### Contextual factors, mental health disorders and psychosocial factors predicting temporal changes in opiate and stimulant use

Multilevel modelling showed that the base model, which only included temporal changes (Time) in drug use, showed a steady and substantial decline over the measurement waves compared to the baseline assessment (Table 3, Model 1). This model also reflected that there was considerable variance to be explained in drug use, and we therefore proceeded to test a second model that included the contextual factors, mental health disorders and psychosocial factors for temporal changes in opiate and stimulant use. As displayed in Table 3 (Model 2), male gender and increased age were associated with a higher risk of use throughout the study period. Further, having a <84 BR score of antisocial personality disorder and having had one or both biological parents with alcohol or drug-abuse problems increased the risk of sustained use of opiate and stimulant use. Changes in work income were not significantly related to changes in drug use during the first two measurement waves compared to baseline, but having work income at the 7-year follow-up was associated with a decreased risk of use. This tendency was also present at 10-year follow-up, albeit not statistically significant. Living with a person who has a substance abuse problem was strongly related to increased risk of drug use across all measurement waves. Participants who received additional inpatient treatment had a significant reduction in use of opiates and stimulants over time, while OMT was rather weakly associated with abstention of opiates and stimulants.

**Table 3:** Temporal multivariate associations between contextual factors, mental health disorders and psychosocial factors with use of opiates and stimulants.

Indicator	Model 1				Model 2			
	AOR	Z	CI 95%	SE	AOR	Z	CI 95%	SE
Intercept	6.47	10.76****		1.12	.52	-.95	.14; 2.00	.36
Time	-	-	-	-	-	-	-	-
Year 0 (ref.)	-	-	-	-	-	-	-	-
Year 1	.09	-11.65****	.06; .13	.09	.11	-6.08****	.05; .22	.04
Year 2	.09	-11.68****	.06; 13	.09	.13	-6.13****	.07; .25	.04
Year 7	.09	-10.86****	.06; .14	.09	.19	-4.62****	.10; .39	.07
Year 10	.06	-10.74****	.03; .10	.06	.10	-4.83****	.04; .25	.05
Age at baseline					1.54	2.22*	1.05; 2.26	.30
Gender (male)					1.04	2.91***	1.01; 1.07	.01
Income from work (yes)					.85	-.37	0.36; 2.01	.37
Primary school problems (yes)					.86	-.78	0.59; 1.25	.16
Education (high)					.79	-1.17	0.54; 1.17	.16
Parent(s) with psychological problems (yes)					.76	-1.44	0.52; 1.11	.15
Parent(s) with alcohol or drug problems (yes)					1.60	2.56**	1.12; 2.30	.30
Antisocial personality disorder (MCMI <84)					1.76	2.97***	1.21; 2.56	.33
Borderline personality disorder (MCMI <84)					1.22	.77	.73; 2.03	.32
Anxiety (MCMI <84)					.86	-.53	.50; 1.49	.24
Dysthymia (MCMI <84)					1.25	.73	.69; 2.26	.38
OMT intermediate (yes)					.19	-3.46****	.07; .49	.09
Inpatient intermediate (yes)					2.93	3.20****	1.52; 5.68	.99
Outpatient intermediate (yes)					1.64	3.35****	1.23; 2.19	.24
Physical abuse (M/H)					.91	-.39	.56; 1.48	.23
Emotional abuse (M/H)					1.06	.25	.65; 1.74	.27
Sexual abuse (M/H)					1.33	1.28	0.86; 2.06	.30
Baseline treatment								
OMT (ref. yes)					-	-	-	-
Inpatient					.64	-1.43	0.35; 1.18	.20
Outpatient					.76	-.75	0.37; 1.56	.28
Living with person who abuses drugs (yes)					1.13	.36	0.58; 2.22	.39
Income from work * Time								
Year 0 (ref. yes)					-	-	-	-
Year 1					.76	-.49	.24; 2.33	.43
Year 2					1.09	.16	.37; 3.21	.60
Year 7					.18	-2.66**	.05; .63	.12
Year 10					.44	-1.12	.11; 1.84	.32
Living with person who abuses drugs * Time								
Year 0 (ref. yes)					-	-	-	-
Year 1					5.46	3.49****	2.10; 14.16	2.66
Year 2					6.07	3.54****	2.24; 16.50	3.10
Year 7					3.44	2.17*	1.12; 10.50	3.44
Year 10					14.86	3.44****	3.19; 69.14	11.66
OMT * Time								
Year 0 (ref. yes)					-	-	-	-
Year 1					4.00	2.42*	1.30; 13.34	2.30
Year 2					3.36	2.17*	1.12; 10.02	1.87
Year 7					2.73	1.68	.85; 8.81	1.63
Year 10					3.60	1.86	.93; 13.95	2.49
Inpatient * Time								
Year 0 (ref. yes)					-	-	-	-
Year 1					.32	-2.55**	0.14; 0.77	.14
Year 2					.19	-3.78****	.08; .45	.08
Year 7					.20	-2.73**	.06; .64	.12
Year 10					.11	-2.81***	.02; .51	.09
*Variance Components*								
Intercept	2.07	-	1.29;3.32	.50	1.66	-	.93; 2.97	.49
Time	.04	-	.02; .09	.02	.06	-	.03; .12	.12
Intercept/time	-.13	-2.04*	-.25; -.01	.06	-.12	-1.70	.-27; .02	.07
*Fit Statistics*								
-2 LL			-1172.61				-1031.06	

****p < .001,

*** p < .005,

**p < .01,

*p < .05

AOR = adjusted odds ratio

SE = standard error

CI = confidence interval

## Discussion

The present study has shown a substantial decrease in opiate and stimulant use in a SUD treatment cohort during a 10-year study period. At baseline, 80% reported use of these drugs last month prior to treatment admission, whereas 34% reported last-month use ten years later. The greatest changes were found for heroin use (74% reduction) and for amphetamine use (44% reduction). Important contextual predictors of sustained use of these drugs were male gender and living with a person who abused drugs. There was a tendency of reduction of use by work income at the two last follow-up waves. Individuals who received additional residential treatment during the observation period had a significant reduction in use. The most important psychosocial factor associated with sustained use was having had one or both biological parents with severe substance-abuse problems, whereas antisocial personality disorder was the strongest predictor of sustained use among the mental health disorders.

In drug classifications (e.g. [21]), heroin and amphetamines are often regarded as two of the drugs with the strongest addictive and physical harm potentials. The most substantial changes found in the current study were among those who used these drugs. The findings showed that heroin had the greatest decline from baseline to 1-year follow-up, and thereafter decreased even further towards the end of the observation period. This aligns with other studies [20], [15], [16], [12], [17] and extend the results to a ten-year follow-up. Our findings are also in line with earlier studies which showed that heroin and amphetamines tend to decline in tandem over time [20], [15].

The prevalence of cocaine use was low (5%) at baseline and did not change significantly during 10 years. The stability of cocaine use complements previous findings (e.g. [29]), while the NTORS study [12] showed that crack cocaine was more than halved for the crack users at 4–5 years from intake, but a quarter of the non-crack users at baseline reported use of this drug during follow-up. The percentage of patients with a primary cocaine problem in Norwegian specialised treatment for ICD-10 diagnosed SUD patients is still very low, with an incidence rate of less than 1% in 2015 [38].

In the current study, we divided predictors associated with opiate and stimulant use in contextual factors, mental health disorders and a psychosocial category. Contextual factors remain rather understudied in the SUD population. In a recent study, Darke et al. reported that contextual factors were relatively weak predictors of drug use in a treatment cohort interviewed 11 years after inclusion [16]. Contradicting this conclusion, we found that living together with a person who abuses drugs during the observation period comprised a severe risk factor for sustained opiate and stimulant use. This was one of the strongest predictors of use when other contextual factors, mental health disorders and psychosocial factors were adjusted for in the multivariate model. The finding draws attention towards the challenges of establishing and keeping drug free relations for many of these patients. It also points at the needs for material conditions amplifying adequate housing.

There were tendencies in the growth model reflecting that the relative importance of living with a person who abuses drugs and, to some extent work income, increased over time. Again, this underlines that drug-free households and income from work are important elements in a long-term recovery process. Our results further seem to reflect that inpatient treatment was associated with abstinence in opiate and stimulant use over time. This finding corresponds with the former studies, ATOS, DATOS and NTORS, and with the aims of most Norwegian inpatient programmes, as one of their main treatment goals is total abstinence from drugs. Further, the patients who take part in this type of treatment are usually older and have more severe drug use problems than outpatients. Both of these factors may promote treatment motivation and retention [39], [40]. Certainly, there are fewer control mechanisms related to outpatient treatment and when measuring use of opiates and stimulants, treatment within these facilities was not associated with abstention. The continuous use of heroin among OMT patients has been described in previous work [41], [6] and our results are in line with those findings.

Among the mental health disorders, we found that indications of antisocial personality disorder (ASPD) were important for sustained use of opiates and stimulants. Several previous studies have shown that ASPD is common in the SUD treatment population and associated with poorer treatment outcomes and prognosis [39], [42]. ASPD is associated with cognitive deficits including high impulsivity and poor emotional regulation that increase drop-out risk and instability [43]. Behaviours connected to these deficits could undermine the establishment and maintenance of drug-free relations needed for family life and work. The fact that 70% of the patients in our cohort are described with one or more personality disorders, and 50–60% with at least one lifetime period of anxiety and depression, reconfirms that these are patients with a high degree of comorbidity and in need of qualified, specialized treatment. Of note, Andreas et al. [3] found a dose-response effect where mental distress increased both in magnitude and over time with the number of drugs used. In the current study the anxiety and dysthymia variables were not significant predictors of change in poly-drug use. Whether these contradictory findings could be ascribed to the operationalization of poly-drugs by opiate and stimulant use in the present study is of interest for further investigation.

Our results point at a factor not described in previous large-scale cohort studies (ATOS, DATOS and NTORS), namely having had one or both biological parents with severe alcohol/drug problems. Finding this as a predictor for sustained use at the end of a 10-year observation period illuminates the importance of identifying such conditions both in preventive measures and in treatment and aftercare interventions. As almost 50% of our cohort reported to have had parents with severe substance-abuse problems, continued research on the complex interplay between genetic and environmental influences on developing and sustaining drug use through generations seems of profound importance.

### Strengths and limitations

The current study included a relatively large cohort of SUD patients with a 10-year follow-up and multiple measurement waves. The instruments used, amplified a broad description of the patient cohort and feasible predictors for testing changes in drug use. Besides, the follow-up rate was high throughout the observation period. However, the study has some limitations that warrant discussion. Our assessments of opiate and stimulant use are based on the last 30 days preceding baseline and follow-up interviews. On the basis of these measurements we cannot generalise the use across the entire observation period. The study describes the use/non-use of heroin, other opiates, amphetamine and cocaine. This analysis yields a feasible indication of opiate and stimulant use, but does not reflect complete information about the spectrum of drug use among the patients. The study information was gathered from patient interviews and self-reports. No biomarkers were collected, and this has to be taken into consideration when interpreting the results, even though self-reported drug use has shown high levels of validity in clinical research settings [44], [9] and the interviewers were part of a research group with no affiliation or conflict of interest with the patients. It could be argued that the observed decline in drug use was due to aging effects in the cohort. However, this explanation is not very plausible in regards of the strongest drug decline observed at the 1- and 2-year follow-ups, as these time intervals are probably insufficient for substantial age effects to occur. The plausibility of this explanation was further reduced because age at baseline was adjusted for as a covariate in the analysis. This covariate was found to have a relatively modest relation to opiate and stimulant use. Meanwhile, age effects may explain some of the observed decline in stimulant and opiate use at the 7- and 10-year follow-ups. Finally, even if the applied instruments allowed for a broad investigation of predictors for temporal changes in drug use, assessment of more externalizing mental health disorders like attention deficit hyperactivity disorder (ADHD) would have been an important supplement. There are also additional personality disorders to antisocial and borderline disorders assessed in the MCMI. However, these two personality disorders were chosen in line with previous literature with the aim of replication over an expanded temporal period.

## Conclusions

The current study has demonstrated a substantial temporal decline in opiate and stimulant use over 10 years in a SUD cohort. These encouraging findings are of importance, as these drugs and the combination of them are known to cause high physical and mental harm as well as comprehensive societal costs. The predictors identified for sustained use warrant a focus towards children growing up in families with severe substance abuse problems and to behaviours and symptoms related to developing personality disorders like ASPD. Moreover, it calls into attention the establishment and maintenance of drug-free relations in the recovery process among patients with SUD and their need for highly qualified services to obtain relational competence. Drug-free households and entering work arenas demands a dualistic interplay between mastering relational and professional skills and a policy of societal integration.

## Supporting information

S1 Datafile Data file. (SAV)
